# EMBRACING CULTURE AND DESIGNING AN INTERGENERATIONAL TRAINING PROGRAM TO SUPPORT LATINE ELDERS

**DOI:** 10.1093/geroni/igad104.1022

**Published:** 2023-12-21

**Authors:** Quinton Cotton, Paul Kissell, Elma Johnson, Yolima Chambers, Manka Nkimbeng, Joseph Gaugler

**Affiliations:** University of Minnesota, Minneapolis, Minnesota, United States; University of Minnesota School of Public Health, Minneapolis, Minnesota, United States; University of Minnesota, Minneapolis, Minnesota, United States; Centro Tyrone Guzman, Minneapolis, Minnesota, United States; University of Minnesota, Minneapolis, Minnesota, United States; University of Minnesota, Minneapolis, Minnesota, United States

## Abstract

Although there have been efforts to promote health equity and increase representational diversity in research, there is a gap in our understanding on how to integrate cultural considerations into the intervention design process. Dementia caregiving interventions too frequently lack prioritization and integration of community-directed and culturally appropriate inputs during intervention planning. Consequently, intervention fit among racial and ethnic populations is sometimes culturally incongruent and intervention acceptability in community settings is weakened. We conducted a formative analysis drawing from research team member memos, document review, World Cafes with Latine community residents, and group interviews with Latine organizational staff to understand the intervention development process of an intergenerational dementia training program to support elders within the Latine community. The design process integrated iterative periods of listening through data gathering, informing by applying learned information to the developing program, and affirming through validation. Our analysis demonstrates the need for a broad range of skills and capacity to facilitate effective intervention design (language mastery among key research team members, program design skills, listening, relationship building).

